# Complete genome sequence of BLRI-developed inactivated avian influenza A/H9N2 vaccine strain in Bangladesh

**DOI:** 10.1128/mra.00304-26

**Published:** 2026-06-25

**Authors:** Mohammed A. Samad, Md. Rezaul Karim, A. S. M. Ashab Uddin, Anowar Hossen, Md. Zulfekar Ali, Md. Mizanur Rahman Khan

**Affiliations:** 1 Genome Sequence Laboratory, Transboundary Animal Disease Research Center, Bangladesh Livestock Research Institute (BLRI) 560480, Savar, Dhaka, Bangladesh; 2 Animal Health Research Division, Bangladesh Livestock Research Institute (BLRI), Savar, Dhaka, Bangladesh; Katholieke Universiteit Leuven, Leuven, Belgium

**Keywords:** complete genome sequence, avian influenza virus, A/H9N2 vaccine, Bangladesh

## Abstract

The H9N2 Avian influenza virus is widely distributed and is responsible for significant economic losses in the global poultry industry. An A/H9N2 inactivated vaccine has been developed from the local circulating strain A/chicken/Bangladesh/NRL-H9-1767/2022(H9N2). The local isolate belongs to the G5.7 lineage. Here, we report the complete genome sequence of the Bangladesh Livestock Research Institute-developed A/H9N2 vaccine strain.

## ANNOUNCEMENT

The avian influenza virus is a segmented, negative-sense RNA virus that belongs to the family of Orthomyxoviridae, genus *Alphainfluenzavirus influenzae* (1, 2). Avian influenza viruses are categorized into different subtypes according to the antigenic variation of the two surface glycoproteins, hemagglutinin (HA) and neuraminidase (NA). Currently, 19 hemagglutinin (HA) subtypes and 11 neuraminidase (NA) subtypes have been identified, based on the antigenic characteristics of these envelope-associated surface proteins (3, 4). The H9N2 low-pathogenic avian influenza virus is endemic in poultry populations across many regions of Asia, the Middle East, and North Africa, leading to substantial economic losses (5). Vaccination is considered an effective strategy for controlling H9N2 infection. An inactivated A/H9N2 vaccine was developed from the locally circulating strain A/chicken/Bangladesh/NRL-AI/1767/2022 (H9N2) by propagation in specific pathogen–free embryonated chicken eggs at 37°C for 48–72 h (6). The vaccine virus was chemically inactivated with beta-propiolactone. This viral isolate was selected based on antigenic cartography analysis (7). In this study, we report the complete genome sequence of the vaccine strain A/chicken/Bangladesh/NRL-H9-1767/2022(H9N2), which was isolated from a chicken farm in Bangladesh in 2022.

Total RNA was extracted from the culture allantoic fluid of A/H9N2 vaccine strain in 9- to 10-day-old embryonated chicken eggs using the Quick-RNA Viral Kit (Cat. No. R1035, Zymo Research), following the manufacturer’s protocol. A sequencing library was prepared using the Illumina Standard Total RNA Prep, Ligation with Ribo-Zero Plus Kit (Illumina, USA), resulting in an average fragment size of 400 bp. Sequencing was performed on the Illumina NextSeq 2000 platform with 2 × 150 bp paired-end chemistry, yielding approximately 2.64 million read pairs (27.71% of the original read set). Raw read quality was assessed using FastQC v0.11.3 (8). Adapter trimming and quality filtering were conducted with Trimmomatic v0.39.2 (9), applying the following parameters: minimum quality score > 30, minimum read length > 35 bp, and 5′ clip of 15 bases for both forward (R1) and reverse (R2) reads. Reads specific to A/H9N2 virus were identified using Kraken2 v2.1.3 (10) in conjunction with the viral reference database (version: k2_viral_20210517). The extracted A/H9N2 reads were assembled *de novo* using SPAdes v3.14.0 with k-mer sizes of 77 and 99 (11), followed by reference-based alignment to the reference sequence from the National Center for Biotechnology Information (NCBI) (KM285394 to KM285401). The complete genome of this H9N2 strain consists of eight single-stranded RNA segments with full lengths (Table 1). Completeness of the genome was confirmed by alignment with the H9N2 complete genome sequence (KM285394 to KM285401) (12), which showed full coverage of all coding sequences as well as the non-coding 5′ and 3′ UTRs, indicating that sequencing extended to both genome ends. Sequence analysis revealed that the nucleotide sequences of the HA and NA genes of the A/chicken/Bangladesh/NRL-H9-1767/2022(H9N2) strain both belong to the G1 lineage based on phylogenetic analysis. The amino acid sequence at the HA cleavage site is PAKSKR↓GLF, which is characteristic of low-pathogenic avian influenza viruses (13). HA amino acid sequence revealed eight potential N-glycosylation sites at positions 29 (NST), 57 (NVT), 74 (NGT), 84 (NVT), 141 (NVT), 218 (NVT), 282 (NVS), and 306 (NVT). Amino acid positions 226 and 228 (H3 numbering) of the HA receptor-binding sites were Q and G, respectively, which are characteristic of avian-adapted influenza viruses and confer preferential binding to α2,3-linked sialic acid receptors (14). The isolate possesses E and D at positions 627 and 701 of the PB2 protein, which is characteristic of viruses of avian origin (15).

**TABLE 1:** Comparison of the nucleotide sequences of the eight gene segments of the isolate A/chicken/Bangladesh/NRL-H9-1767/2022(H9N2) with its closest relatives in GenBank^a^

Gene^a^	GenBank accession no.	Depth of coverage	Nucleotide size (bp)	GC content (%)	Most closely related virus strain (GenBank accession no.)	Sequence identity (%)
PB2	PQ824445	13,600×	2,341	43.53	A/chicken/Bangladesh/NRL-BLRI-H9-330-PB2/2024 PQ834361	100
PB1	PQ824446	13,700×	2,339	43.06	A/chicken/Bangladesh/NRL-BLRI-H9-330-PB1/2024 PQ834362	100
PA	PQ824447	24,300×	2,232	43.31	A/chicken/Bangladesh/NRL-BLRI-H9-330-PA/2024 PQ834363	100
HA	PQ824448	11,300×	1,741	42.78	A/chicken/Bangladesh/NRL-BLRI-H9-330-HA/2024 PQ834364	99.77
NP	PQ824449	23,000×	1,565	43.87	A/chicken/Bangladesh/NRL-BLRI-H9-3037-NP/2022 PQ834373	100
NA	PQ824450	22,500×	1,466	43.19	A/chicken/Bangladesh/NRL-BLRI-H9-330-NA/2024 PQ834366	99.93
M	PQ824451	45,600×	1,072	43.38	A/chicken/Bangladesh/NRL-BLRI-H9-3065-MP/2022 PQ834565	100
NS	PQ824452	15,300×	890	42.97	A/chicken/Bangladesh/NRL-BLRI-H9-330-NS/2024 PQ834368	100

^a^ Nucleotide sequences from all eight gene segments of A/chicken/Bangladesh/NRL-H9-1767/2022.

**Fig 1:**
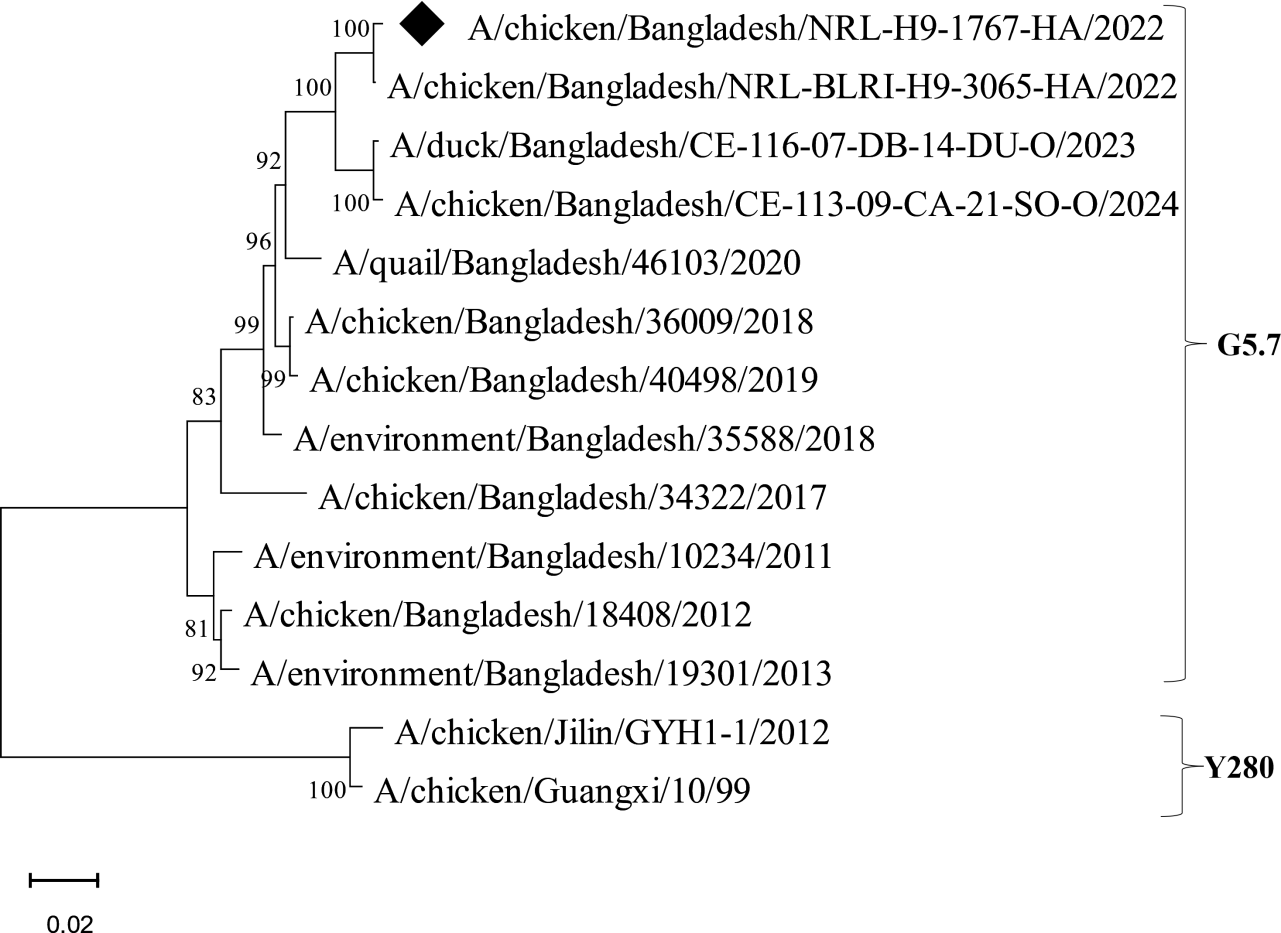
Phylogenetic tree of HA gene segment of A/H9N2 vaccine strain. Phylogenetic analysis was conducted using nucleotide sequences. Reference strains were selected based on geographic relevance and lineage representation, and sequence alignment was performed using the ClustalW algorithm implemented in MEGA version 11 with 500 bootstrap values. Bar, 0.02 nucleotide substitutions per site. The sequence from this study is depicted with a black diamond.

Sequence similarity was determined using BLASTn against the NCBI GenBank database, which is depicted in Table 1. According to phylogenetic analysis, the HA genes of the A/H9N2 vaccine strain in this study belonged to the G5.7 lineage, with a bootstrap value of 100% (Fig. 1).

## Data Availability

The complete genome sequence of the A/H9N2 vaccine strain in Bangladesh has been submitted to GenBank under accession numbers PQ824445, PQ824446, PQ824447, PQ824448, PQ824449, PQ824450, PQ824451, and PQ824452. The raw sequence reads have been submitted to the Sequence Read Archive database under accession number SRR37289099.
